# Acute pulmonary histoplasmosis of immunocompetent subjects from Martinique, Guadeloupe and French Guiana: a case series

**DOI:** 10.1186/s12890-023-02388-6

**Published:** 2023-03-22

**Authors:** Moustapha Agossou, Jean-Marie Turmel, Aude Aline-Fardin, Nicolas Venissac, Nicole Desbois-Nogard

**Affiliations:** 1 Department of Respiratory Diseases, CHU of Martinique, Route de Chateauboeuf, 97200 Fort-de-France, France; 2 Department of Infectious Diseases, CHU of Martinique, Fort-de-France, France; 3 Department of Pathology, CHU of Martinique, Fort-de-France, France; 4 Department of Thoracic Surgery, CHRU of Lille, Lille, France; 5 Department of Parasitology-Mycology, CHU of Martinique, Fort-de-France, France

**Keywords:** Acute histoplasmosis, Pulmonary histoplasmosis, Acute respiratory failure, *Histoplasma capsulatum*

## Abstract

**Introduction:**

Histoplasmosis is a fungal disease caused by *Histoplasma capsulatum*. *Histoplasma capsulatum* var *capsulatum* is found in Martinique.

Cluster cases following working in deserted house, have been described in Martinique. Cases of acute pulmonary histoplasmosis have been described in immunosuppressed individuals, or in case of substantial exposure to reservoirs of *Histoplasma capsulatum*; however, cases of acute histoplasmosis are rare in immunocompetent individuals.

**Cases series:**

We report a series of 4 cases of sporadic acute pulmonary histoplasmosis in immunocompetent subjects. Investigation revealed definite exposure in one patient and 3 cases with potential exposure. The diagnosis was microbiological and histological in 3 patients and histological in one patient. All subjects had positive serology to histoplasmosis. Pulmonary involvement was in the form of nodules and micronodules in 3 cases and ground glass lesions in one case. Patients were treated with itraconazole for 3 months and all had a favourable outcome.

**Conclusion:**

We report a series of 4 cases of acute pulmonary histoplasmosis in immunocompetent individuals, occurring in a context where exposure was uncertain. This raises the problem of occult exposure in the Caribbean. Interventions to raise awareness and encourage caution are warranted targeting the population of the French West Indies and French Guiana.

## Background

Histoplasmosis is a disease caused by a fungus of the genus *Histoplasma capsulatum*. Two varieties cause infections in humans, namely *Histoplasma capsulatum* variety (var.) *capsulatum*, and *Histoplasma capsulatum* var. *duboisii*.

*H. capsulatum* var *capsulatum* is endemic in warm, humid regions of North America (Ohio and the Mississippi river valley), Latin America, Africa and Asia [1–3]. Several cases of histoplasmosis have been described in the Caribbean region, including the French West Indies, French Guiana and Reunion Island [4–6].

Pulmonary involvement is the most common form, since it is the site of primary infection. Disseminated forms are mainly described in immunocompromised subjects [2, 7, 8]. Natural reservoirs of conidia include bat guanos and bird droppings [9].

Cases of sporadic pulmonary histoplasmosis are rare in Martinique in immunocompetent individuals, and most cases reported to date involved clusters occurring after massive exposure during cave explorations [4, 10]. One epidemiological study performed in Martinique found an incidence of 0.34 cases per 100,000 inhabitants, 80% of which involved HIV (human immunodeficiency virus) immunocompromised subjects [11]. Only one pulmonary involvement was noted in this series of 10 patients and concerned an immunocompromised subject.

We report a series of 4 cases of acute histoplasmosis in immunocompetent subjects from Martinique, in the French West Indian territories of the Caribbean.

## Case presentations

### Case 1

**Fig. 1:**
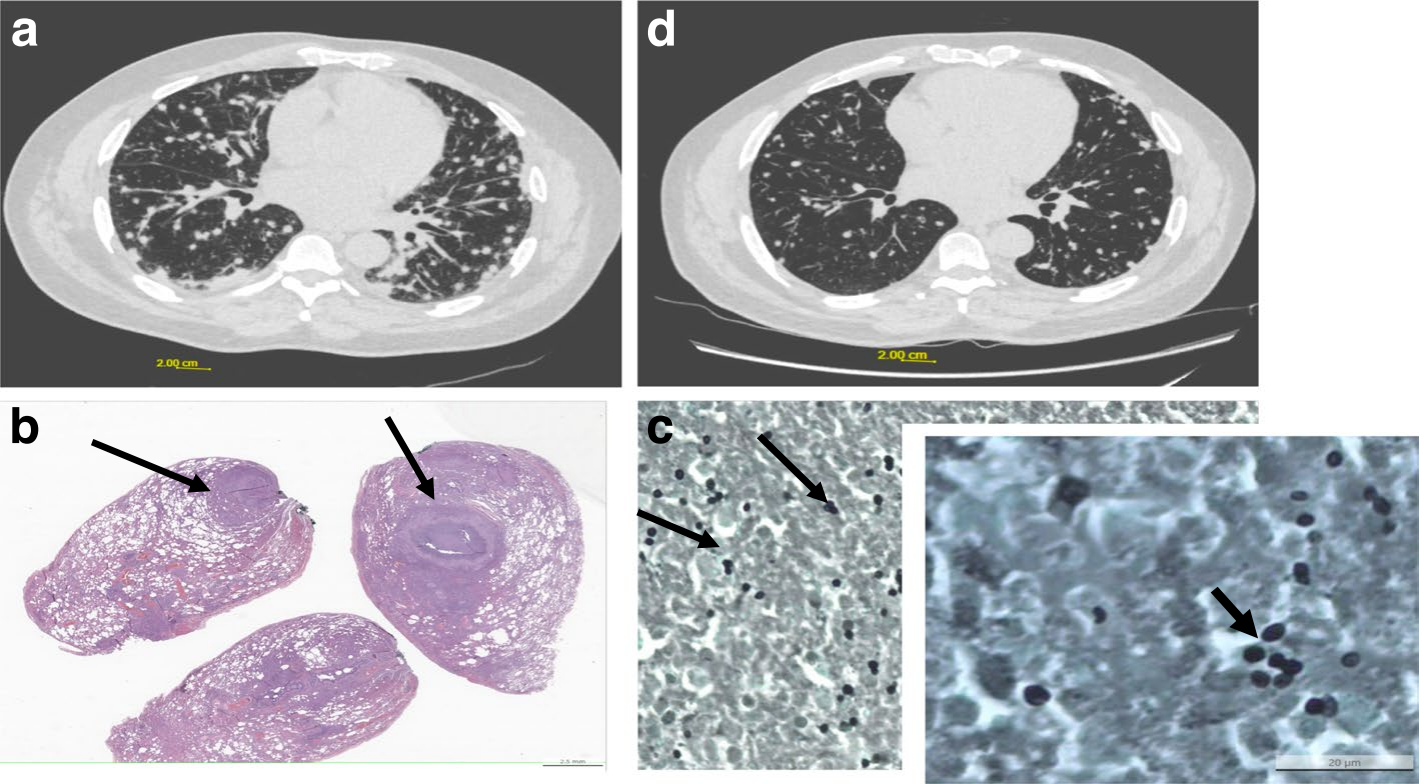
**a**: initial chest CT scan of case 1 showing diffuse and sometimes confluent nodules and micronodules. **b**: suppurative circumscribed necrosis and giant and epitheloïd cell. **c**: Small yeasts in black on Grocott stain. **d**: 3-month follow-up CT scan of case 1 showing lung abnormalities improvement

Case 1 was a 56-year-old male patient with no notable prior medical history, except active smoking, estimated at 40 pack-years. He worked as a journalist and raises snakes and mice as a hobby. He had no history of recent travel or known recreational exposure to *Histoplasma*. The patient consulted his general practitioner (GP) for febrile cough lasting for a week. He reported an evening fever, and the cough was dry. Symptoms persisted after empirical antibiotic therapy with amoxicillin/clavulanic acid for 7 days. He developed dyspnea, and a chest X-ray was performed, which showed diffuse pulmonary nodules and micronodules. The patient was then referred to the emergency room for suspected tuberculosis in May 2019. Clinical examination on admission found blood pressure of 102/59 mmHg, body temperature of 39.4 degrees Celsius, heart rate 90 beats per minute, and oxygen saturation of 97% in room air. There was no weight loss and no haemoptysis, but significant asthenia. The chest was clinically clear on auscultation. Biology results (see Table 1) showed negative HIV serology, and serum protein electrophoresis was normal. The computed tomography (CT) scan performed in the emergency room showed multiple bilateral disseminated pulmonary nodules, some appearing excavated and associated with mediastino-hilar adenomegaly (Fig. 1a).

**Table 1:** Clinical, biological, therapeutic, and outcome data of 4 cases of acute pulmonary histoplasmosis in immunocompetent individuals

No	Age (years)	Medical history	Reasons	Identifiable exposure	Serology	PCR BAL	BAL	Polynuclear eosinophils in G/L (%)	Radiological aspect	Histology, Pulmonary Biopsy	Treatment	
											Molecules	Duration
1	56	Smoking, 40 pack years	Febrile cough	No	Positive	Positive	Negative culture Cytology: 1,250,000 elements/mm^3^ 56% Lymphocytes CD4/CD8 0.73	0.12 (1)	Diffuse nodules and micronodules (Fig. 1a)	Positive	Amphotericin B	2 weeks
											Itraconazole	3 months
2	61	None	Febrile dyspnea	Yes, works as a carpenter	Positive	Positive	Negative culture Cytology:510,000 elements/mm^3^ 29% lymphocytes CD4/CD8 to 2.12	0.36 (4.3)	Diffuse ground glass image and mediastinal lymphadenopathy (Fig. 2a)	Positive	Itraconazole	3 months
3	52	None	Febrile dyspnea	Yes: works as a market gardener	Positive	Not done	Negative culture	0.07 (0.8)	Diffuse nodules and micronodules (Fig. 3a)	Positive	Itraconazole	3 months
4	68	None	Fever	Yes: ground drilling	Positive	Positive	Positive culture	0 (0)	Diffuse pulmonary nodules and micronodules Fig. 4a)	Not done	Itraconazole	3 months

#### Sputum acid-fast bacillus (AFB) test was negative, and the Quantiferon-TB® Gold (QTF-G) test was also negative

In the absence of diagnostic certainty and given the worsening of symptoms, particularly dyspnea, quadruple therapy against tuberculosis was started. A pulmonary surgical biopsy was performed, and the histological analysis revealed an abundant polymorphic and granulomatous inflammatory infiltrate with giant cells and a necrotic center. The necrosis was inflammatory, suppurative and non-caseating. PAS (periodic acid-Schiff) and Grocott staining revealed the presence of numerous small (3-4µ) yeast-like pathogens, grouped in clusters within the necrosis (Fig. 1b and 1c). Ziehl staining did not show any mycobacteria.

Mycological direct examination with May Grunwald Giemsa (MGG) coloration, and mycological culture on Sabouraud’s medium, prolonged for 3 months were negative. *Histoplasma capsulatum* RT-PCR (reverse transcriptase-polymerase chain reaction) was performed according to the technique described by Alanio et al. [12], and was found to be positive on the surgical biopsy. The histoplasma serology was positive. To rule out pulmonary aspergillosis, aspergillus serology was performed and was negative. The main characteristics of the patient are described in Table 1. Acute pulmonary histoplasmosis was diagnosed. Treatment with liposomal amphotericin B for 15 days was started, followed by itraconazole 200 mg daily for 3 months.

Clinical improvement was observed with this treatment. The follow-up CT scan also showed radiological improvement with partial regression of all bilateral disseminated pulmonary micronodules and mediastino-hilar adenomegaly (Fig. 1d).

### Case 2

**Fig. 2:**
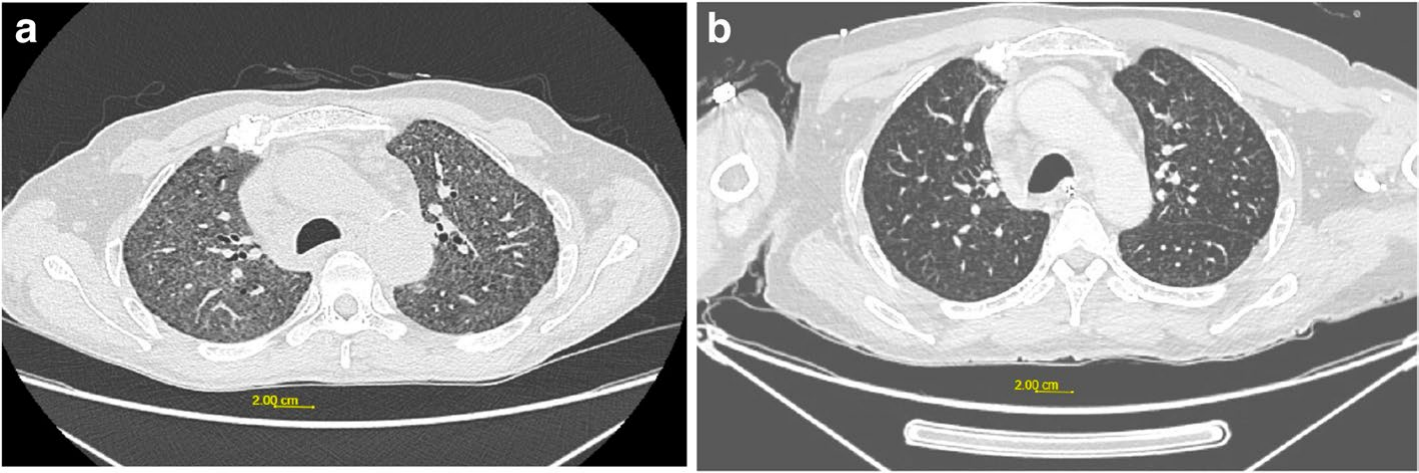
**a**: Initial chest CT scan of case 2 showing diffuse ground glass lesions. **b**: 6-month follow-up CT scan showing improvement of ground glass lesions

Case 2 was a 61-year-old patient with no specific medical history, non-smoker, working as a carpenter in French Guiana since February 2019. He consulted his GP who referred him to the emergency room in July 2019 for dyspnea, dry cough and altered general condition lasting for about two weeks. The examination revealed hypoxemia with a saturation of 82% requiring 3L/min of oxygen. The baseline assessment showed a C-reactive protein (CRP) of 37 mg/L (Limits 0–5 mg/L), full blood count parameters were within normal limits, and D-dimers at 0.75 µg/mL (limit 0–0.5 µg/mL). The patient underwent a thoracic CT angiography, which did not reveal pulmonary embolism, but showed diffuse infiltrative lung disease with diffuse ground-glass lesions (Fig. 2a). Miliary tuberculosis was suspected. Sputum AFB was negative. The patient then underwent bronchial fibroscopy with bronchoalveolar lavage. There were no endobronchial abnormalities. Mycological, bacteriological and mycobacteriological examinations were negative. The patient was referred to our center for further management after the initiation of presumptive tuberculosis treatment.

Clinical examination found blood pressure of 99/66 mmHg, body temperature of 36.4 degrees Celsius, heart rate of 103 beats per minute, respiratory rate of 24 cycles per minute, and oxygen saturation of 96% under 2L/min oxygen. Pulmonary auscultation found a reduction of vesicular sounds. The biological workup (detailed in Table 1) showed negative HIV serology, while histoplasmosis serology by detection of antibodies, using *sebia/Meridian-IELP Immunoelectrophoresis, Histoplasma IELP*, was positive with 2 precipitation arcs (normal = 0 precipitation arcs). PCR testing for *Histoplasma capsulatum* according to the method described above was positive.

In view of the pulmonary findings, mimicking hypersensitivity pneumonitis, we ruled out Farmers’s lung and bird fancier's lung, with negative serologies. The findings were also suggestive of non-specific interstitial lung disease in a context of auto-immune disease. In the absence of relevant clinical signs, the auto-immune work-up found anti-nuclear antibodies at 1/160 with a speckled appearance and anti-Midbody antibodies. Anti-soluble nuclear antigen antibodies, Antineutrophil Cytoplasmic Antibodies, rheumatoid factor and Anti Cyclic Citrulline Peptides antibodies, and myositis antibodies were within reference intervals. Aspergillosis serology was negative.

Thoracic CT scan showed diffuse centrilobular micronodules with ill-defined contours, predominantly in the upper lobes, and right paratracheal adenomegaly of 15 mm in the minor axis (Fig. 2a). We noted the presence of calcified pulmonary nodules and liver calcifications. Bronchial fibroscopy showed no endobronchial abnormalities. A mediastinoscopy was performed and enabled the removal of a right laterotracheal adenopathy (2R). Histological examination revealed numerous yeasts on Grocott staining, suggesting *Histoplasma capsulatum* var *capsulatum* (Fig. 1c). Mycological direct examination with MGG coloration and mycological culture on Sabouraud’s medium were negative. The main characteristics of the patient are detailed in Table 1.

Treatment with itraconazole 200 mg per day was initiated for 3 months. Clinical and radiological outcome was favourable, with a decrease in ground glass lesions (Fig. 2b). There were no abnormalities in the lung parenchyma after 2 years.

### Case 3

Case 3 was a 52-year-old patient, working as a market gardener, who consulted the emergency room in June 2020 for dyspnea that had been progressively worsening for 2 weeks. He had no notable medical history. Two years previously, he had enriched his crop fields with bat droppings. He had taken protective measures for dung recovery and soil enrichment, but continued to work the land without protective measures.

**Fig. 3:**
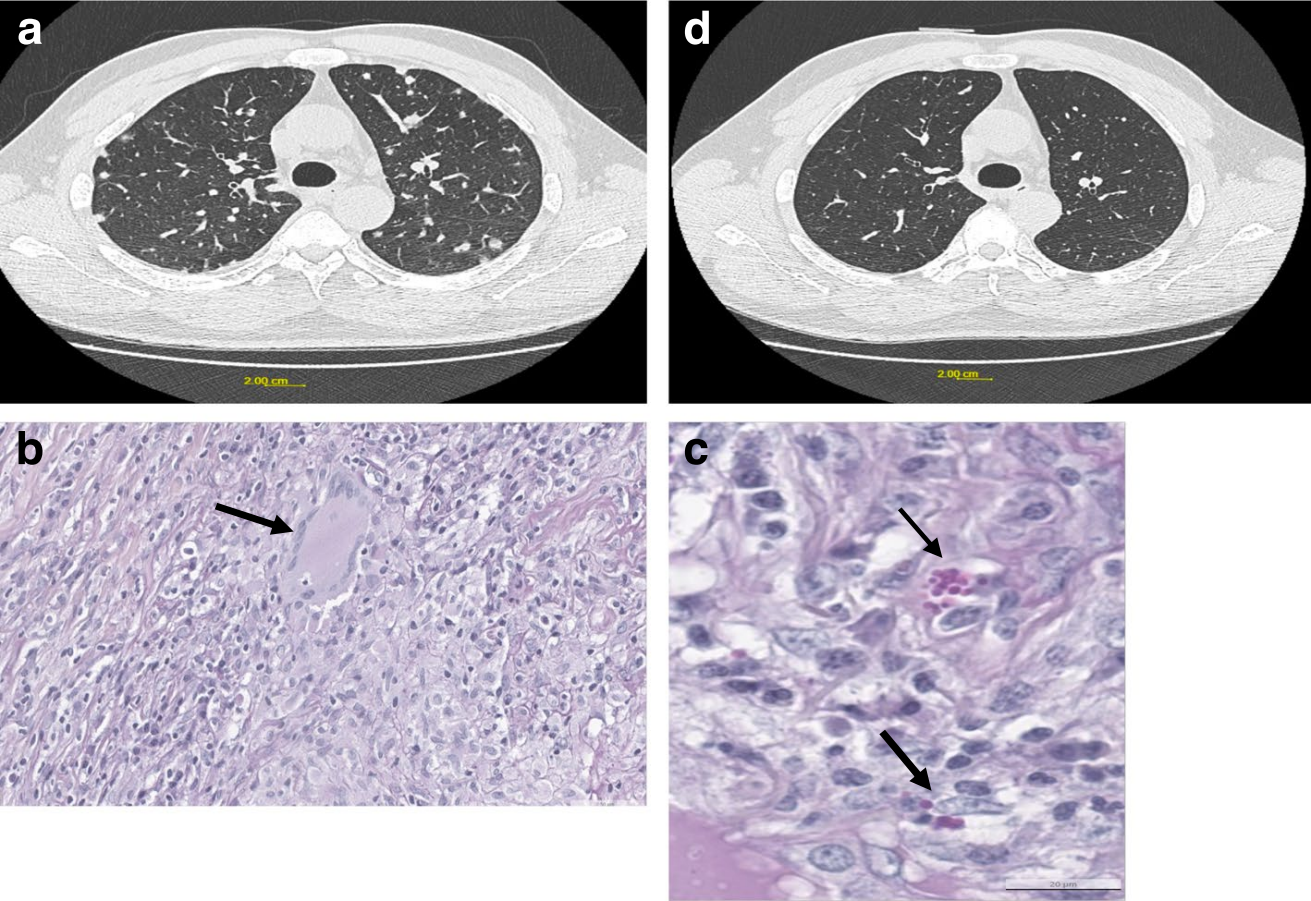
**a**: Initial chest CT of case 3 showing bilateral pulmonary nodules. **b**: Picture show a giant cell. **c**: Small yeasts in pink on Periodic Acid Schiff stain. **d**: Control scan at 3 months with improvement of the pulmonary anomalies in case 3

Clinical examination found fever at 38° Celsius, blood pressure of 122/74 mmHg, heart rate at 113 beats per minute, respiratory rate of 32 cycles per minute, oxygen saturation 94% in room air. The chest was clinically clear on auscultation. In terms of biology (Table 1), HIV serology was negative. Serum protein electrophoresis revealed an oligoclonal pattern involving IgG and IgM kappa and lambda. The thoracic CT angiography did not find pulmonary embolism, but revealed multiple diffuse bilateral micronodular lesions of about 6 mm (Fig. 3a). Sputum AFB was negative.

Bronchial fibroscopy was performed and found no endobronchial abnormality. The bacteriological examination of the bronchoalveolar lavage (BAL) was negative. Direct examination and culture on Sabouraud agar were negative. Cytology was also negative. The detailed characteristics of the patient are described in Table 1.

A surgical lung biopsy was performed. Histological examination found inflammatory granulomatous epithelioid and giganto-cellular changes, with necrosis and the presence of yeast-like structures, suggestive of histoplasmosis (Fig. 3c and 3c. In addition, histoplasmosis serology was positive, and galactomannan antigen testing was negative. Itraconazole treatment at a dose of 200 mg daily was prescribed. Clinical and radiological outcome was favourable (Fig. 3d).

### Case 4

**Fig. 4:**
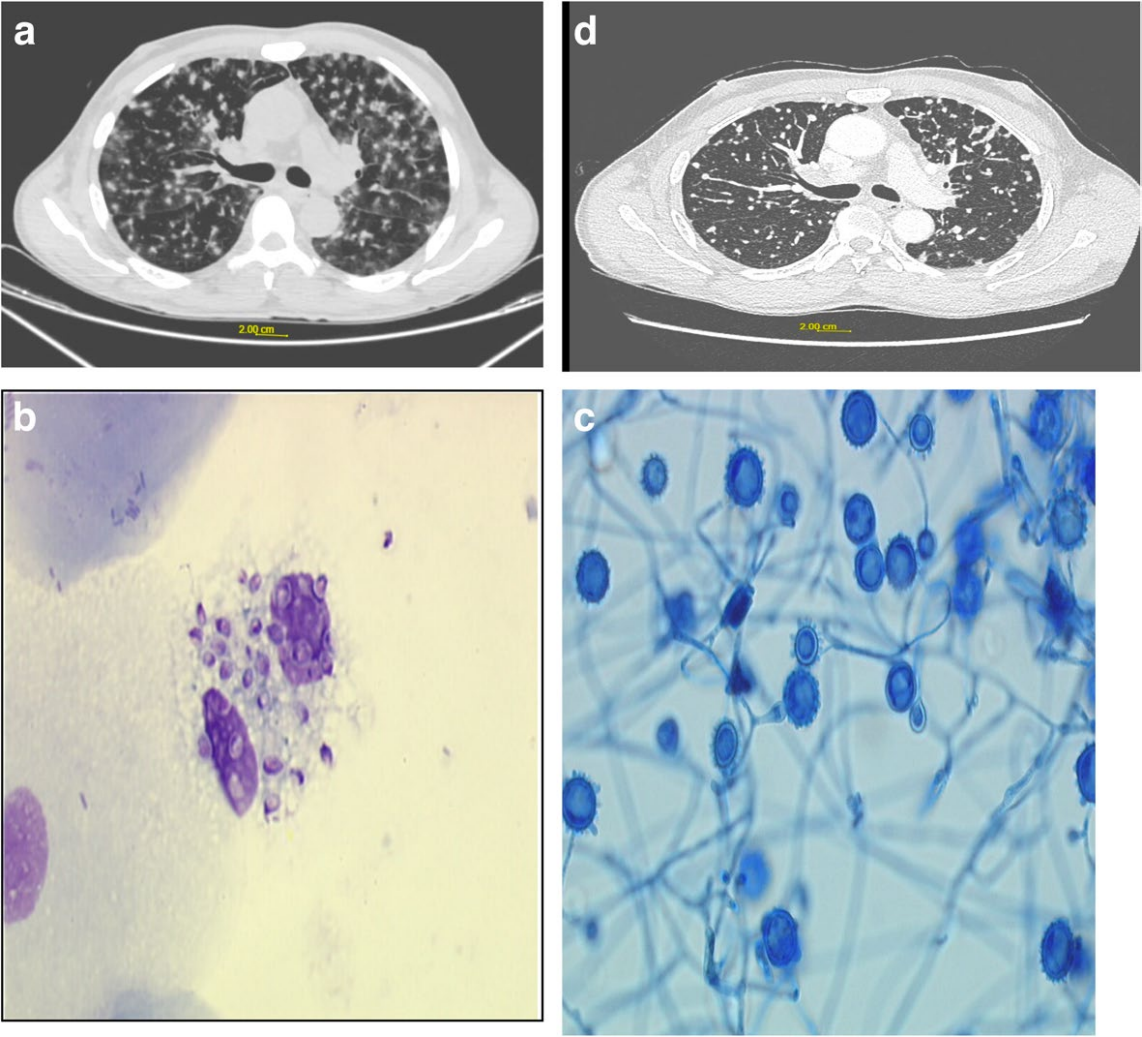
**a**: Initial chest CT of case 4 showing pulmonary nodules and micronodules sometimes confluent. **b**: Microscopic examination (X100) of a *Histoplasma capsulatum* var. *capsulatum* with MGG coloration. **c**: Microscopic examination (X400) of a *Histoplasma capsulatum* var. *capsulatum* culture on Sabouraud's medium. **d**: CT scan at 6 months in case 4 showing slight improvement but persistence of nodular lung images despite clinical improvement

Case 4 was a 68-year-old male patient, with no notable medical history. He was drilling for a company that worked changing telephone poles, and he stayed in Guadeloupe from January 15 to February 06, 2021. He had physical asthenia associated with diffuse arthromyalgia with fever and loss of taste, but without anosmia. On February 6, the patient returned to Martinique, but the fever persisted, with the appearance of anorexia and episodes of diarrhea, prompting the patient to present to the emergency room. Clinical examination revealed blood pressure 132/79 mmHg, fever at 39.1° Celsius, heart rate of 59 beats per minute, and oxygen saturation of 95% in room air. Auscultation of the lung revealed rales in both bases. The patient’s characteristics are described in Table 1. Chest CT scan (Fig. 4a) showed a small bilateral pleural effusion, non-compressive mediastino-hilar adenomegaly and diffuse micronodular and nodular infiltration of random distribution, associated with ground glass areas. Sputum was negative for AFB and culture was negative for MTB. No endobronchial abnormality was found on fibroscopy. The bronchoalveolar lavage was negative for AFB and culture was negative for MTB. RT-PCR SARS-CoV 2 (severe acute respiratory syndrome coronavirus 2) was negative. The mycological examination revealed very rare yeasts of small size (2–5 µ) with MGG coloration, suggestive of *Histoplasma capsulatum* var *capsulatum* (Fig. 4b). Culture on Sabouraud’s medium revelled many colonies of *Histoplasma capsulatum* var *capsulatum* (Fig. 4c).

Histoplasmosis serology was positive with the presence of 1 precipitation arc. The Beta D-glucan assay was 110 pg/mL (Normal < 80 pg/mL). Galactomannan antigen was negative. Treatment with itraconazole 200 mg daily for 3 months was initiated. Outcome in clinical and radiological terms was favourable, albeit slow (Fig. 4d).

## Discussion

Histoplasmosis is an invasive fungus that most often affects the lungs due to its mode of acquisition. The acute form is often described in immunocompromised subjects or in case of massive exposure [13–16]. The disseminated form is mainly described in immunocompromised subjects.

In the Island of Martinique in the French West Indies, clusters of acute lung disease have been described in people working in desert house colonised by bats, and after exploration mountain tunnel full of bats [4, 10].

We report here a series of 4 cases of acute pulmonary histoplasmosis in immunocompetent subjects. All were men, with no notable medical history, presenting for the most part with febrile dyspnea. Three patients were found to have potential exposure: one was a carpenter, one worked in drilling for utility poles, and the third was a market gardener who had enriched his soil with bat droppings. The fourth case reported had no clearly identifiable exposure. Given that men are often affected, there may be occult exposure, or an activity that is in advertently exposing these individuals to the infectious conidia. A predominance of males was also noted in the series by Garsaud et al. [11].

In Martinique, these 4 cases were diagnosed in 2 years. Although Martinique is an endemic area for histoplasmosis, most cases are related to massive exposure [4, 10]. In 2020, a similar case was described by colleagues in Guadeloupe [17].

The patients presented acute dyspnea, or subacute febrile dyspnea. Acute pulmonary histoplasmosis is most often associated with mediastinal and hilar adenopathies. Qing et al. described such findings in a series of 10 cases [18], while Hoenigl et al. observed mediastinal and hilar adenopathy in 2 cases out of 3 in a cluster in Australia [19]. In our series, 3 of the 4 patients had mediastinal and/or hilar adenopathy.

Diagnostically, direct microbiological evidence was available in 3 out of the 4 cases: by mycological culture in 1 case, and by molecular biology in 3 cases. The diagnosis was also supported by positive serology in all patients. Identification of the yeast on histopathological or cytological specimens or in culture at room temperature is the gold standard for diagnosis. According to the European Organization for Research and Treatment of Cancer and the Mycoses Study Group Education and Research Consortium Consensus, the diagnosis can made with evidence of environmental exposure, a compatible clinical illness and the presence of histoplasma antigen in any body fluid [20]. The culture has a low sensitivity in the acute and sub-acute forms [21]. The role of PCR and serology remains unclear among the diagnostic tools for histoplasmosis [20]. Serologic testing appears to have moderate sensitivity [22]. Conversely, PCR performs well for diagnosis of histoplasmosis, with sensitivity of 98%, specificity of 99% and positive and negative predictive values of 82% and 99% respectively [12].

In all cases, the outcome was favourable after antifungal treatment. In severe cases, liposomal amphotericin B can be used, but itraconazole provides a satisfactory clinical response.

This case series suffers from limitations inherent to clinical case descriptions. The epidemiological situation warrants continued monitoring. These cases illustrate the possibility of acute pulmonary histoplasmosis in an immunocompetent individual and without massive exposure to guano.

## Conclusion

Acute pulmonary histoplasmosis is rarely encountered in the immunocompetent except following exposure to heavy inocula of Histoplasma conidia. Our series emphasizes the need for a high index of suspicion of this clinical entity in an endemic setting in the absence of severe environmental exposures.

## Data Availability

The datasets analyzed during the current study are available from the corresponding author on reasonable request.

## Abbreviations

AFB: Acid fast bacillus
CRP: C-reactive protein
HIV: Human immunodeficiency virus
MGG: May grunwald giemsa
PAS: Periodic acid schiff
RT-PCR: Reverse transcriptase-polymerase chain reaction
